# Interstitial Lung Disease in Rheumatoid Arthritis: A Practical Review

**DOI:** 10.3389/fmed.2022.837133

**Published:** 2022-05-13

**Authors:** Antonella Laria, Alfredo Maria Lurati, Gaetano Zizzo, Eleonora Zaccara, Daniela Mazzocchi, Katia Angela Re, Mariagrazia Marrazza, Paola Faggioli, Antonino Mazzone

**Affiliations:** ^1^Asst Ovest Milanese–Rheumatology Unit, Magenta Hospital, Milan, Italy; ^2^Asst Ovest Milanese–Internal Medicine Department, Cuggiono Hospital, Milan, Italy; ^3^Asst Ovest Milanese–Internal Medicine Unit, Legnano Hospital, Milan, Italy

**Keywords:** rheumatoid arthritis, lungs, ILD, bronchiectasis, IPAF

## Abstract

Rheumatoid arthritis (RA) is a systemic inflammatory disease, which primarily causes symmetric polyarthritis. An extrarticolar involvement is common, and the commonly involved organ is lungs. Although cardiac disease is responsible for most RA-related deaths, pulmonary disease is also a major contributor, accounting for ~10–20% of all mortality. Pulmonary disease is a common (60–80% of patients with RA) extra-articular complication of RA. Optimal screening, diagnostic, and treatment strategies of pulmonary disease remain uncertain, which have been the focus of an ongoing investigation. Clinicians should regularly assess patients with RA for the signs and symptoms of pulmonary disease and, reciprocally, consider RA and other connective tissue diseases when evaluating a patient with pulmonary disease of an unknown etiology. RA directly affects all anatomic compartments of the thorax, including the lung parenchyma, large and small airways, pleura, and less commonly vessels. In addition, pulmonary infection and drug-induced lung disease associated with immunosuppressive agents used for the treatment of RA may occur.

## Introduction

Rheumatoid arthritis (RA) is a systemic inflammatory disease that primarily causes symmetric polyarthritis. Nevertheless, extra-articular involvement is common, and lungs are affected in 60–80% of cases (1, 2). Although heart disease is largely responsible for RA-related mortality, pulmonary disease is also a major contributor, accounting for 10–20% of all-cause deaths. RA may affect all anatomic compartments of the thorax, including lung parenchyma, large and small airways, pleura, and less commonly pulmonary vessels (3, 4). Airway infections and drug-related pulmonary toxicity associated with an immunosuppressive therapy may further add up and complicate the picture (3, 4). Optimal screening, diagnostic, and treatment strategies of RA-associated pulmonary diseases are still unmet and represent the subject of an ongoing investigation. Clinicians should carefully assess patients with RA with symptoms and on the other side consider RA and other connective tissue diseases when evaluating a patient with pulmonary disease of an unknown etiology (1, 2). This aspect is very important given the recent introduction of specific antifibrotic therapies such as nintedanib and pirfenidone, whose efficacy and safety for patients with RA are up-to-date under investigation.

## Epidemiology and Risk Factors

Respiratory diseases particularly interstitial disease (RA-ILD) and bronchiectasis (BR) are common extra-articular manifestations of RA, with an estimated prevalence between 10 and 30% (5, 6), depending on the population analyzed and the imaging methodology used for detection. The probability of developing interstitial lung disease (ILD) in patients with RA is higher compared to controls and increases over time with ILD occurring typically within 5 years of RA diagnosis. In up to 20% of patients, it may even precede joint disease (7, 8). Whereas, RA is typically more common in women, RA-ILD is more frequently detected in men, with a male-to-female ratio of 2:1 (9). Remarkably, patients with RA-ILD have a 3-fold increased risk of premature death compared to patients with RA without ILD (7, 10), with a median survival of 3 years following ILD diagnosis (7). The association between RA and BR has long been recognized. The prevalence of BR in RA was originally estimated around 2–12% based on clinical symptoms; however, subclinical BR might have a prevalence of up to 30–50% as more recently reported in studies using a high-resolution computer tomography (HRCT) (11). Coexistence of BR and RA is associated with a higher mortality compared to BR alone (12). A number of studies report that age older than 65 years, smoking habit, and male gender have been recognized as the risk factors for developing RA-ILD (13–15). Moreover, specific RA characteristics, such as serum positivity for anti-citrullinated protein/peptide antibodies (ACPA) (16, 17), and/or the rheumatoid factor (RF) (13, 18), the presence of the RA-associated human leukocyte antigen HLA-DR4, and the presence of other extra-articular manifestations, in particular subcutaneous nodules, were found to be significant predictors for ILD development [(6, 19, 20); Table 1].

**Table 1:** Predictors of Interstitial Lung Disease (ILD) development and severity in RA patients.

- Age > 65 years old
- Male gender
- High RF levels
- ACPA positivity
- Subcutaneous nodules
- HLA-DR4 (DRB1*04) haplotype
- Smoking habit
- FVC <60% and DLCO <40%
−6–12 month worsening in FVC of ≥10%, or in DLCO of ≥15%
- UIP pattern
- ≥20% of the lungs affected
- Diagnostic delay>24 months


## Etiopathogenesis

**Figure 1:**
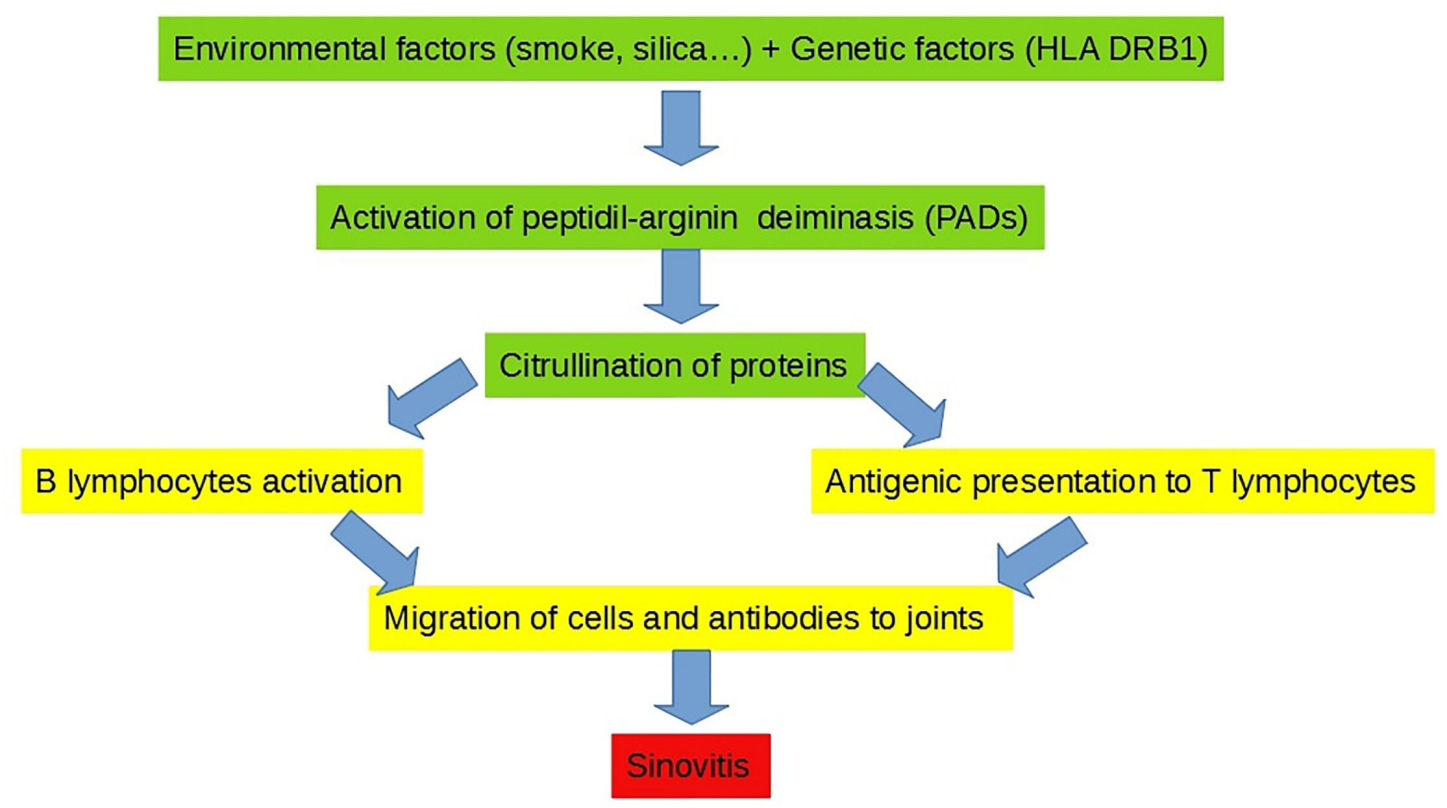
Lungs as a site for the initiation of rheumatoid arthritis (RA).

Pathogenesis of pulmonary changes in the established RA may involve various cell types and subtend complex interactions among different cell compartments. Pulmonary manifestations in RA are probably triggered by both local and systemic insults. Environmental factors, such as smoking or an exposure to other inhalants (e.g., mineral dusts in Caplan's syndrome) on one hand and systemic and vascular inflammations on the other hand, may synergize together and provoke alveolar inflammation and interstitial fibrosis (21). Recent findings support a crucial role of the lungs as key mucosal sites in which a loss of immune tolerance results in the generation of RA-specific autoimmunity that precedes joint disease and clinical arthritis onset (21). Indeed, one of the main etiopathogenetic hypotheses that is emerged in recent years is that in the presence of a genetic predisposition (e.g., HLA-DRB1 haplotype; male sex), external factors (e.g., cigarette smoke, silicone, or other allergens) may induce the aberrant citrullination of alveolar peptides and proteins, which in turn triggers the activation of T and B lymphocytes, leading to the production of ACPA autoantibodies and immune complexes, thus ultimately eliciting peripheral arthritis (21). Therefore, in RA, lungs are primarily involved as extra-articular sites in the initiation of the disease and secondarily involved as extra-articular sites of the established disease (21) (Figure 1).

## Clinical Pictures

**Figure 2:**
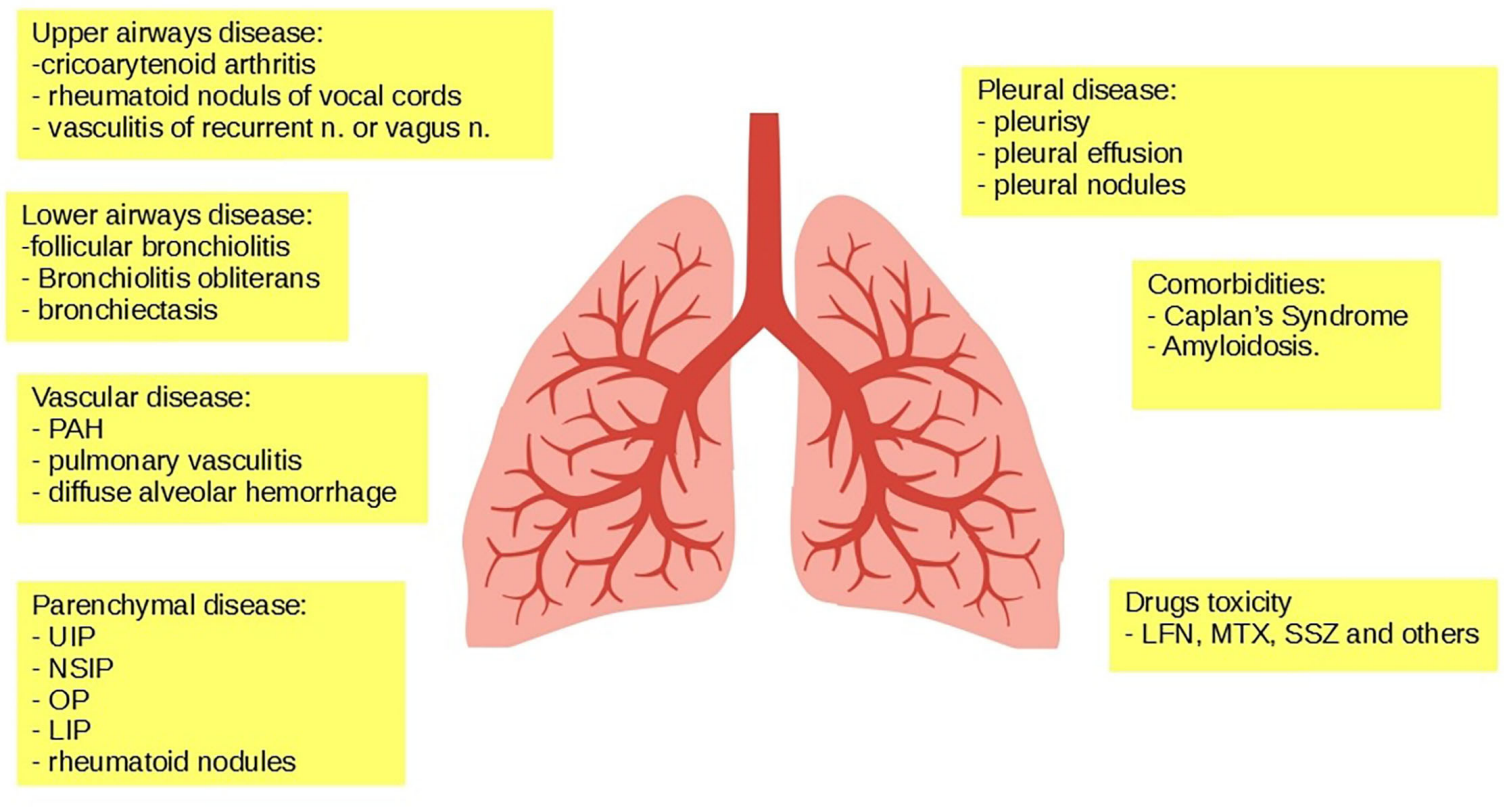
Respiratory involvement in RA.

The respiratory system can be less often involved at the upper level, with rare cases of cricoarytenoid or cricothyroid arthritis, rheumatoid nodules affecting the vocal cords, and vagus nerve or recurrent black laryngeal vasculitis (22–25). Lower airways are instead affected in up to 65% of the cases, in the form of follicular bronchiolitis (26), obliterative bronchiolitis (27), and/or BR (28). RA can also affect the pleura, manifesting itself as pleurisy or pleural effusion in up to 20% of the cases (29), and more rarely as pleural rheumatoid nodules (30, 31). Lung vasculopathy may account for pulmonary arterial hypertension in almost 20% of the cases (32, 33), pulmonary vasculitis in around 8% of the cases (34, 35), and hemorrhagic alveolitis or diffuse alveolar damage in rare cases (36). The most frequent form of pulmonary involvement in RA is parenchymal disease, which occurs in up to 30% of patients, in the form of either ILD (36–38) or rheumatoid nodules (39). Furthermore, RA has been described in association with pneumoconiosis (following an exposure to asbestos, coal, or silica dusts) (40) and combined with secondary amyloidosis (following arthritis-related chronic inflammation) (41). Drug-associated pulmonary toxicity and hypersensitivity pneumonitis may rarely occur (42, 43) (Figure 2).

## Interstitial Lung Disease

**Figure 3:**
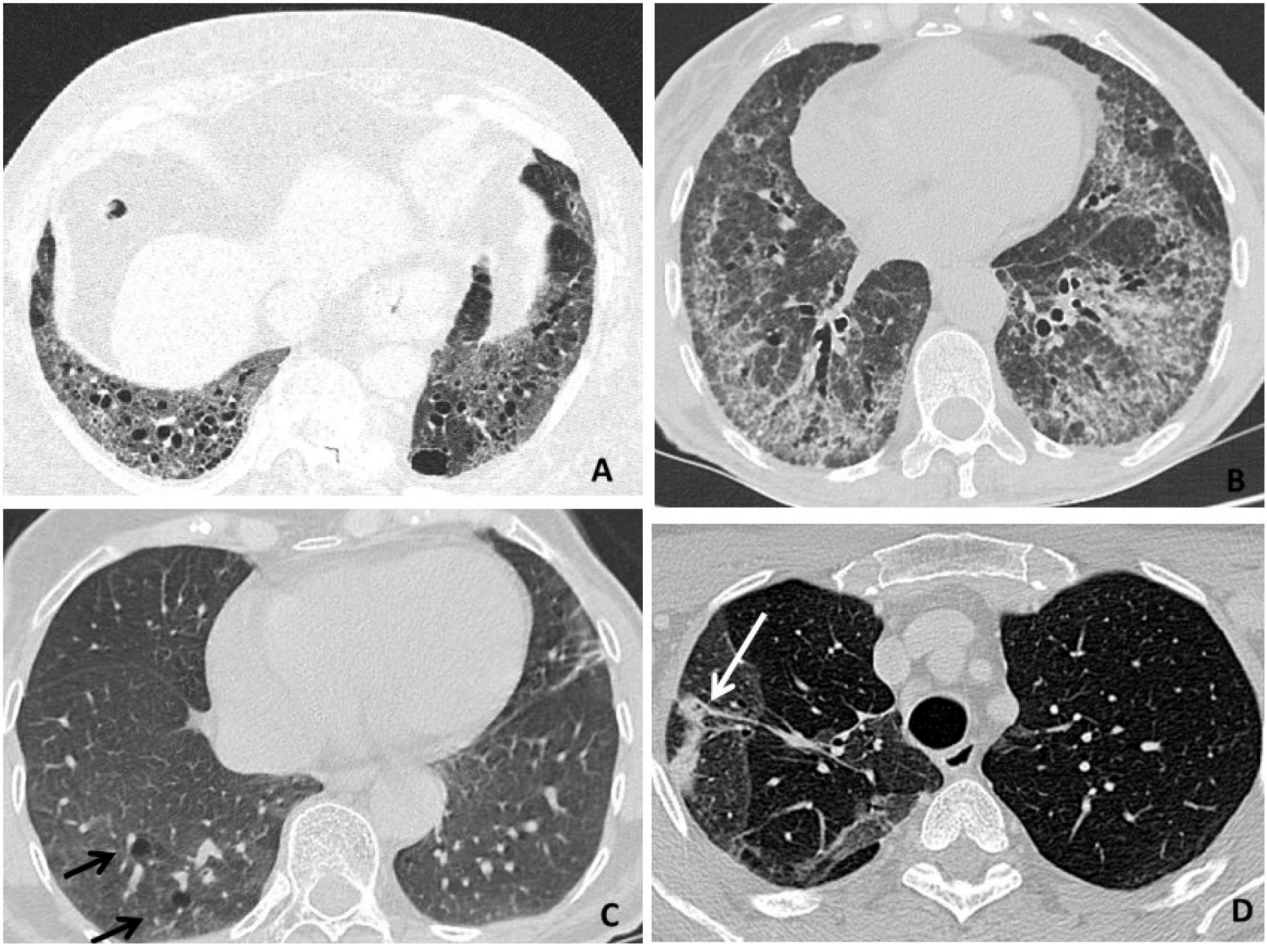
High-resolution computer tomography (HRCT) findings in respiratory diseases and particularly interstitial disease (RA-ILD). **(A)** An usual interstitial pneumonia (UIP) pattern (representing the most typical radiological presentation in patients with RA) is primarily characterized by reticular fibrosis, traction BR, and subpleural cysts (honeycombing). **(B)** A nonspecific interstitial pneumonia (NSIP) pattern is primarily characterized by ground-glass opacities, variably mixed with septal thickening, and reticular fibrosis (fibrosing NSIP). **(C)** A lymphocytic interstitial pneumonia (LIP) pattern is primarily characterized by perivascular thin-walled cysts (black arrows). **(D)** An organizing pneumonia (OP) pattern is primarily characterized by pulmonary consolidations (a white arrow).

Interstitial lung disease is characterized by alveolar inflammation and fibrosis of the pulmonary interstitium. Patients with RA are approximately nine times more likely to develop ILD as compared to the general population (38). HRCT is more sensitive than X-ray in detecting ILD and allows to identify ILD even at subclinical stages (44). The most typical radiological pattern of RA-ILD is usual interstitial pneumonia (UIP), representing more than half of the cases, followed by nonspecific interstitial pneumonia (NSIP), organizing pneumonia (OP), and lymphocytic interstitial pneumonia (LIP) (45, 46). At HRCT, a definite UIP shows a bibasilar and subpleural distribution, with septal thickening, reticular fibrosis, traction BR and bronchiolectasis, and subpleural cysts (the so-called “honeycombing”) (47). NSIP is primarily characterized by bilateral, peripheral, and patchy ground-glass opacities, typically sparing subpleural regions, and variably mixed with reticular fibrosis (i.e., cellular *vs*. fibrosing NSIP) (47). OP is characterized by parenchymal consolidations with air bronchograms and surrounding ground-glass opacities, often bilateral and confluent, with a patchy or lobar distribution (47) (Table 2, Figure 3). An UIP pattern is particularly frequent in patients who are older, men, and had a history of smoking. It confers a worse prognosis, with survival rates similar to those seen in idiopathic pulmonary fibrosis (48, 49, 52, 53) and more frequent hospitalization rates for respiratory exacerbations compared to patients with other HRCT patterns (50). ILD is usually observed early in the natural history of RA (54), and may even precede the onset of articular symptoms in one-fifth of cases (8, 51, 55, 56). As previously mentioned, up to nearly half of patients with RA may have a subclinical ILD on HRCT (57, 58). Patients with RA-ILD commonly report nonspecific respiratory symptoms, such as dry cough and exertional dyspnea, which might be confused with fatigue or arthritis-related functional impairment (51, 59–61). Less frequent symptoms include chest pain, wheezing, and productive cough in the presence of traction BR and concomitant infections (62).

**Table 2:** HRCT findings of Interstitial Lung Disease in RA (ILD-RA).

UIP – Usual Interstitial Pneumonia	13–56%	Bilateral, basilar, subpleural fibrosis Volume loss and architectural distortion Presence of subpleural cysts (“Honeycombing”) Traction bronchiectasis/ bronchiolectasis	Ref (48, 49)
NSIP - Nonspecific interstitial pneumonia	12–30%	Bilateral, symmetric, basilar, peripheral ground-glass opacities Traction bronchiectasis Intra and interlobular septal thickening and consolidation. Tipical of NSIP pattern is subpleural sparing	Ref (48, 49)
OP- Organizing pneumonia	11–15%	Airspace consolidation, often bilateral, usually patchy but can be lobar Sometimes, airspace consolidation can be nodular. Surrounding ground glass opacities Area of involvement can change over time	Ref (50, 51)
LIP- Lymphocytic interstitial pneumonia	Rare	Perivascular thin-walled cysts Can have surrounding ground glass or centrilobular nodules Septal/bronchovascular thickenin	Ref (48, 49)

## Rheumatoid Pulmonary Nodules

**Figure 4:**
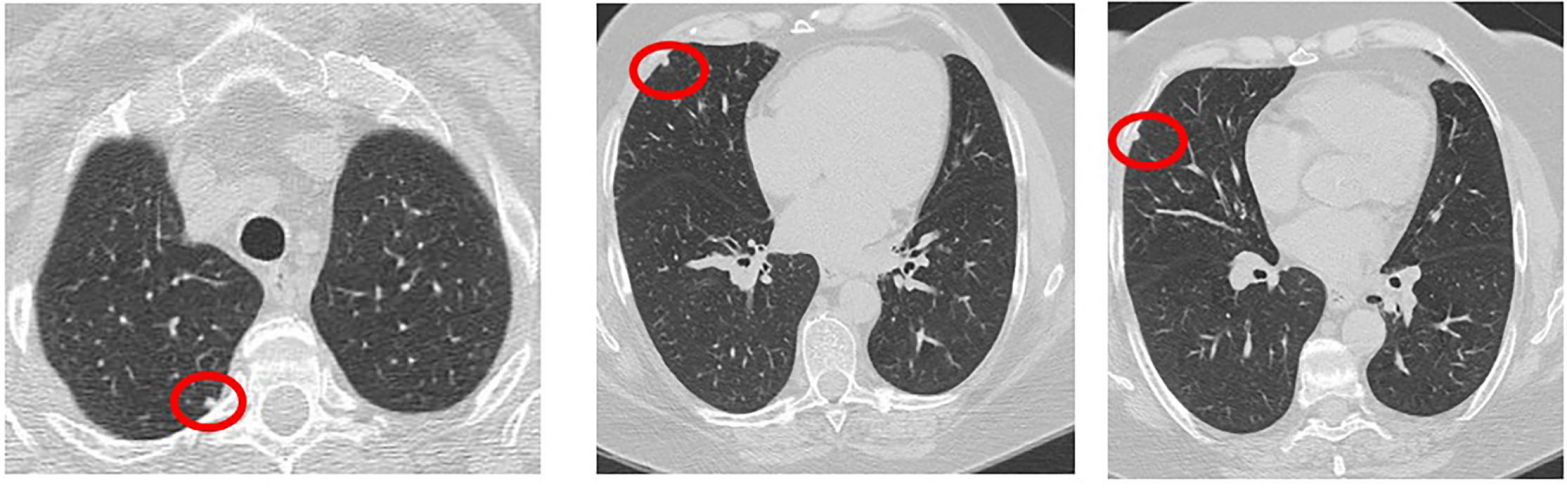
Multiple pulmonary nodules on HRCT in a patient with RA.

Rheumatoid pulmonary nodules occur in about one-third of patients with RA (63). They are mostly asymptomatic and may be resolved spontaneously. Male gender, smoking habit, and RF positivity are well-recognized risk factors (34). Patients with pulmonary nodules are more often younger and may concomitantly have subcutaneous nodules (64). Certain drugs used in the treatment of RA, including methotrexate (MTX), leflunomide, and TNF inhibitors (TNFi), may cause or worsen pulmonary nodulosis and should be then discontinued in such cases (65). Histopathological examination shows granulomatous inflammation with epithelioid cells and chronic inflammatory infiltrates. Fibrinoid necrosis can lead to parenchymal cavitations (34), while necrotizing nodules can result into hemoptysis and pneumothorax following their rupture into the pleural cavity. Rheumatoid pulmonary nodules show distinctive radiological and metabolic characteristics compared to malignancies. CT features include multiplicity (generally ≥4 nodules), smooth borders, cavitations, satellite nodules, peripheral location, pleural contact, and subpleural rinds of soft tissue (Figure 4). Optimal sensitivity (77%) and specificity (92%) for rheumatoid pulmonary nodules can be obtained in the presence of ≥3 CT features (64). Key 18FDG-PET/CT features include low-level metabolism (SUVmax 2.7 ± 2 vs. 7.2 ± 4.8, *p* = 0.007) and the lack of 18F-fluorodeoxyglucose- (FDG-) avid draining lymph nodes (64).

## Bronchiectasis

**Figure 5:**
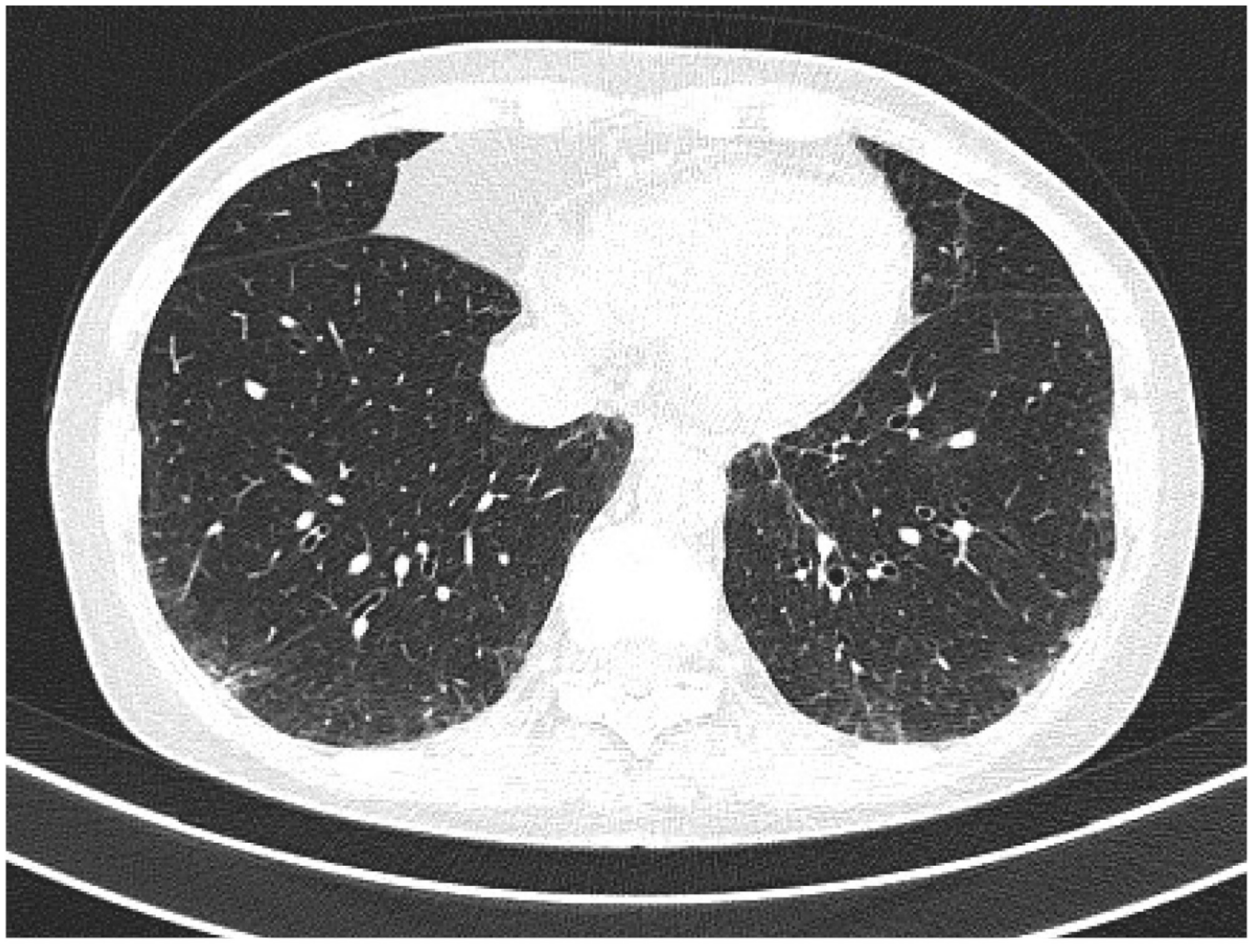
Bronchiectasis (BR) on HRCT in a patient with RA.

Bronchiectasis, defined as irreversibly damaged (66) and dilated bronchi, is one of the most common respiratory manifestations of RA (25, 67). In these patients, the broncho-arterial ratio is typically >1 (Figure 5). BR should be suspected in the presence of chronic cough, sputum production, or recurrent respiratory infections. The diagnosis should be confirmed by HRCT (66, 68). The recognized risk factors for RA-associated BR are: RF and/or ACPA serum positivity, the presence of HLA-DRB1^*^0401 or DQB1^*^0601, DQB1^*^0301, DQB1^*^0201, DQA1^*^0501 haplotypes, and cystic fibrosis transmembrane conductance regulator mutations (68). Management includes a multimodal treatment approach, encompassing pulmonary rehabilitation programs, prophylactic antibiotics, inhaled corticosteroids, and long-acting β_2_-agonists, while the appropriateness of immunosuppressive therapeutics should be carefully evaluated taking into account the high susceptibility to respiratory infections (68).

## Therapeutics

So far, due to limited data, there are no recognized international guidelines for the treatment of RA-ILD (69). In the absence of precise recommendations, moderate-to-severe lung disease in patients with RA should be managed in collaboration with a pulmonologist.

## Steroids

Data on the efficacy of glucocorticoids in the UIP are somewhat controversial: steroids appear to stabilize the lung function in some studies, while others emphasize their increased infectious risk (52, 70, 71). The first proposed guidelines for RA-ILD were those of the British Thoracic Society, dating back to 2008 (72). In these guidelines, the first-line treatment of RA-ILD involves the use of prednisone 0.5 mg/kg/ day for 1–3 months, subsequently tapered up to 10 mg/day or less, possibly combined with a disease-modifying antirheumatic drug (DMARD). In case of steroid failure, the addition of an immunosuppressant, such as cyclosporine, azathioprine, and cyclophosphamide, is recommended (72). Of note, high doses of steroids should be used for inflammatory subtypes of RA-ILD with acute or subacute presentation (i.e., cellular NSIP and OP), but not in fibrotic subtypes typical of advanced and chronic forms (i.e., fibrosing NSIP and UIP) (72, 73).

## MTX and Common DMARDs

Methotrexate has been associated with hypersensitivity pneumonitis and MTX-induced pneumonia (MIP) (74). MIP mostly appears during the first month of therapy, without any predictive factor being recognized (74). Unlike RA-ILD, the onset of MIP is acute or subacute rather than chronic, and common HRCT patterns are NSIP and OP instead of UIP. Moreover, whereas RA-ILD is characterized by tissue neutrophilia, MIP is typically characterized by peripheral and bronchoalveolar eosinophilia. In contrast with a quite poor prognosis of RA-ILD, the prognosis of MIP is overall good, with a complete recovery being observed after a congruous MTX washout period (74). The potential relationship between the use of MTX and the development of RA-ILD has been recently questioned. Juge et al. recently found an inverse association between the use of MTX and the appearance of ILD; in particular, MTX was not associated with an increased risk of RA-ILD, and ILD was detected later in patients with RA treated with MTX (75). In the work of Kiely et al., the time to onset of ILD was later in patients with RA exposed to MTX and the survival curve was higher in a subgroup of patients treated with MTX compared to patients naïve to MTX (76). Indeed, a dose-dependent beneficial effect of MTX on the risk of developing RA-ILD has been demonstrated (77) (Table 3). The Warrick global score (WGS) was significantly lower in patients treated with a MTX dosage ≥ 15 mg/week as compared with patients treated with MTX <15 mg/week or patients naïve to MTX (77). As exposed earlier, in fact, RA-ILD and MIP are distinct entities, with MTX being causative of MIP but protective against the development of RA and RA-ILD; therefore, the detection of RA-ILD would not necessarily imply the discontinuation of MTX.

**Table 3:** Conventional synthetic, biological and targeted synthetic disease-modifying antirheumatic drugs for RA-ILD.

**Drug**	**Contraindicated**	**Possible use but lower beneficial effect on lung**	**Beneficial or stabilizing effect**	**Dubious effects**	**Unknown data**
Steroids			x		
Hydroxycloroquine					x
Leflunomide	x				
Methotrexate		x			
Mycophenolate mofetil		x			
Cyclosporine		x			
Tacrolimus		x			
Cyclophosphamide		x			
Azathioprine		x			
Sulfasalazine	x				
TNFi				x	
Tocilizumab		x			
Abatacept			x		
Rituximab			x		
Anakinra				x	
Anti-JAKs			x		

The role of other disease-modifying DMARDs, such as mycophenolate mofetil (MMF), cyclophosphamide, azathioprine, cyclosporine, and tacrolimus, in the treatment of RA-ILD remains unclear. In a retrospective analysis of 125 patients with CTD-ILD treated with MMF (*n* = 18 RA-ILD), the lung function was improved for those with an NSIP pattern and remained stable for those with an UIP pattern (78) (Table 3). Both MMF and cyclophosphamide have shown an efficacy in systemic sclerosis-associated ILD in double-blind randomized controlled trials (RCTs) (79, 80), whereas there are no RCTs for cyclophosphamide in RA-ILD. Despite limited data, cyclophosphamide is largely used in clinical practice, especially in cases of rapidly progressive RA-ILD. MMF is considered as the main alternative to cyclophosphamide as both induction and maintenance therapy, by virtue of its lower toxicity. Azathioprine may be used as an alternative to MTX when drug toxicity is suspected. A single-center retrospective cohort study on patients with CTD-ILD (*n* = 97, 24% RA-ILD) found that patients treated with azathioprine had similar clinical events and longitudinal pulmonary function tests (PFTs) compared to those treated with MMF (81). There are only small series and few case reports showing an improved lung function in patients with RA-ILD treated with cyclosporine or tacrolimus (82, 83). Of note, sulfasalazine has been associated with hypersensitivity pneumonitis, with nearly half of the patients presented with pulmonary infiltrates and eosinophilia; clinical improvements usually occur following drug discontinuation, yet progressing respiratory failure and the cases of death have also been described (84, 85). Respiratory manifestations associated with sulfasalazine include NSIP, OP, granulomatous disease, bronchiolitis obliterans, and pleural effusion (86, 87). Leflunomide has also been variably associated with rapid-onset hypersensitivity pneumonitis and a new onset or the progression of pre-existing ILD (87, 88). No data have been so far available regarding the potential pulmonary toxicity of hydroxychloroquine (84) (Table 3). Therefore, although some of these drugs can be effective in controlling RA-ILD, potential pulmonary toxicity and low efficacy of certain molecules on articular disease should be also considered (89, 90).

## TNFi and Others bDMARDs

Several cases of newly detected or exacerbated ILDs upon the treatment with TNFi have been reported (91, 92); however, so far, data are overall inconclusive. In a retrospective cohort study of the British Society for Rheumatology Biologics Register, the use of TNFi in RA-ILD was not associated with higher mortality as compared with conventional synthetic DMARDs (csDMARDs) (93). Analyses of large US administrative claim databases did not find statistically significant differences in the risk of respiratory events in patients with RA-ILD using TNFi as compared with abatacept, rituximab, or tocilizumab (94, 95). However, there were numerically fewer respiratory events among patients treated with abatacept compared to TNFi (96). Moreover, a trend toward better survival was observed for rituximab as compared to TNFi (97). A comprehensive search on the PubMed, Embase, Ovid, Cochrane, China National Knowledge Infrastructure, and Wanfang database was performed from their inception to November 2018 (98). In total, 7 original articles and 28 case reports were eligible for an analysis. All 7 cohort studies demonstrated the lack of benefit from TNFi treatment in patients with ILD; indeed, TNFi could be associated with pulmonary adverse events (Table 3). Case reports further suggested such negative findings, showing that TNFi was harmful in 87.5% of the cases and even increased mortality (99). By contrast, small and uncontrolled studies have broadly shown that the majority of RA-ILD patients treated with abatacept, rituximab, or tocilizumab remained stable or improved, as assessed by PFTs and HRCT (99, 100) (Table 3). In an observational multicenter study, 263 patients with RA-ILD were treated with abatacept alone or combined with MTX or another csDMARD (101). It emerged that abatacept is effective and safe in the treatment of RA-ILD, and is associated with high retention rates (around 75%). All 3 treatment groups experienced the stabilization or improvement of respiratory items, namely dyspnea severity, forced vital capacity (FVC), diffusing capacity for carbon monoxide (DLCO), and HRCT findings, as well as the improvement of articular disease activity score assessed on 28 joints (DAS28) (101). A significantly stronger steroid-sparing effect was observed for abatacept in combination with MTX or another csDMARDs compared to abatacept alone. In an Italian multicenter retrospective study, 44 patients with RA-ILD were treated with abatacept for at least 6 months; abatacept appeared to be safe, and FVC, DLCO, and HRCT remained stable in 77.8%, 58.3%, and 70.4% of patients, respectively (102). A recent systematic review including one case series and eight observational studies confirmed the efficacy and safety of abatacept in RA-ILD (103). After a mean follow-up of 17.4 and 47.8 months, the improvement or stabilization of FVC or DLCO was observed in over 85% of the cases, while the improvement or stabilization of ILD imaging was observed in 76.6% and 92.7% of the cases, respectively, regardless of the radiological pattern and more remarkably in patients with a shorter lung disease duration. Abatacept led to a significantly lower probability of ILD worsening compared to TNFi and csDMARDs, being associated with a 90% reduction in the relative risk of lung function deterioration at 24 months (103). A Portuguese, retrospective, multicenter cohort study assessed the response to rituximab in patients with CTD-ILD, of whom 24 had RA-ILD. At 12 months, DLCO and FVC values ameliorated, and particularly promising results with rituximab were obtained in the presence of an NSIP pattern (104). A total of 28 patients with RA-ILD treated with at least one dose of tocilizumab were retrospectively collected in an Italian multicenter study, demonstrating a good safety profile of tocilizumab in these patients and its potential role in lung disease stabilization (105). After a mean follow-up of 30 months, indeed, both FVC and DLCO remained stable in 56%, while HRCT findings were stable in 89% of patients (105).

## Jak Inhibitors

Data regarding the possible roles of Jak Inhibitors in the treatment of RA-ILD are limited. In RA clinical development programs of tofacitinib and baricitinib, 0.1% of patients newly developed ILD (106); however, tofacitinib was not associated with ILD exacerbation (106). In a multicenter observational study, 47 patients with RA-ILD and 387 patients with only RA (without ILD) were treated with tofacitinib. Retention rates were similar for patients with RA-ILD or only RA, and, in most of the patients, PFTs remained stable during a follow-up (107) (Table 3).

## Pirfenidone and Nintedanib

Currently, two anti-fibrotic agents were approved by US Food and Drug Administration (FDA) for the management of IPF, namely nintedanib and pirfenidone. Nintedanib is a small-molecule inhibitor of the tyrosine kinase receptors of platelet-derived growth factor (PDGFR) α and ß, fibroblast growth factor (FGFR) 1-3, and VEGFR 1-3; it also inhibits lymphocyte-specific protein tyrosine kinase, Lyn protein tyrosine kinase, proto-oncogenic protein tyrosine kinase Src, and colony stimulating factor receptor-1 (108). To date, only nintedanib has been studied in a double-blinded RCT in patients with progressive fibrosing ILD, including the cases of RA-ILD. Nintedanib was found to reduce the FVC decline originally in IPF and subsequently in systemic sclerosis-associated ILD (109, 110). Following these results, the INBUILD trial (NCT02999178), an international, double-blind RCT comparing nintedanib to placebo was conducted in patients with progressive fibrosing lung disease (with a baseline extension >10% on HRCT) of different types (of whom 13% were RA-ILD) (111). In this study, progression was defined as: a relative decline in the FVC of at least 10% of the predicted value; a relative decline in the FVC of 5– <10% of the predicted value plus the worsening of respiratory symptoms or an increased extent of fibrosis on HRCT; or the worsening of respiratory symptoms plus an increased extent of fibrosis on HRCT despite the treatment rather than nintedanib or pirfenidone (111). Patients treated with nintedanib had a significantly slower FVC decline over 52 weeks although no significant difference in subjective symptoms or clinical events was observed. In particular, the results were similar to those observed in IPF, with a between-group FVC difference of 107.0 ml/ year in favor of nintedanib (95% CI, 65.4–148.5; *p* < 0.001) (111). The results were significant irrespective of a HRCT pattern. Among the 663 patients included in the INBUILD trial, 170 patients had CTD-ILD, of whom 89 had RA-ILD. A subgroup analysis confirmed significant results for patients with CTD-ILD, with a between-group FVC difference of 104.0 ml/ in favor of nintedanib (95% CI, 21.1–186.9; *p* < 0.41 for the treatment by subgroup by time interaction) (112). Diarrhea was the major side effect of nintedanib, occurring in two-third of the treated patients, which led to a dose reduction in one-third of patients and drug discontinuation in one-fifth of cases (112). The results from the INBUILD trial led FDA to approve nintedanib for the treatment of chronic fibrosing ILDs with a progressive phenotype, including RA-ILD forms^1^ (Table 3). Notably, nintedanib is the first drug approved for this indication. Ongoing trials on RA-ILD include: APRIL (NCT03084419; abatacept vs. placebo); TRAIL1 (NCT022808871; pirfenidone vs. placebo); PULMORA (NCT04311567; tofacitinib vs. MTX); EvERR-ILD (NCT02990286; rituximab with MMF vs. placebo); and RITUX-IP (NCT02251964; rituximab) (113). The management of comorbidities is critical for RA-ILD outcomes. Chronic obstructive pulmonary disease (COPD) frequently accompanies RA-ILD even among non-smoker patients, and a close adherence to the global initiative for COPD management is therefore required in these cases (114). Concomitance with gastroesophageal reflux disease (GERD) is also common, occurring in about half of patients with RA-ILD (115). Causal relationships between GERD and ILD are still a matter of debate. Equally controversial is whether proton pump inhibitors may in turn increase the risk of pneumonia (116). Pharmacological (e.g., proton pump inhibitors, H2 blockers) and non-pharmacological interventions (e.g., weight loss, dietary modification, and raising the head of the bed) are frequently prescribed in ILDs and conditionally recommended in IPF management guidelines (117). Balancing ill-defined risks and the benefits of antacid use in RA-ILD would certainly deserve further investigation.

## Suggested Algorithm for the Assessment, Monitoring, and Management of RA-ILD

The monitoring of treatment response in RA-ILD involves the assessment of the activity and severity of both articular and respiratory disease. Baseline evaluation of pulmonary involvement should include clinical examination, PFTs, the identification of radiological patterns, and the assessment of disease extension by HRCT (118, 119). Clinical examination should also include arterial oxygen saturation and a 6-min walking test (120). Reduced walking distance and oxygen desaturation below 88% are, in fact, known poor prognostic factors in IIPs (117, 120). PFTs should be performed in all patients with RA with respiratory symptoms. Baseline FVC <60% of the predicted values and DLCO <40% of the predicted values are independent predictors of early death in patients with IIPs (121). Importantly, a 6–12 month decline in FVC of at least 10% and/or a decline in DLCO of at least 15% is associated with increased mortality in patients with IIP (96, 122). HRCT imaging is indicated in patients with respiratory symptoms or in clinically asymptomatic patients with a DLCO <70% of the predicted values (59). Individuals with HRCT findings consistent with an UIP pattern (i.e., basal dominant honeycomb cysts with little or no ground-glass changes) and high fibrotic scores have a worse prognosis compared to those with HRCT features indicative of other types of IIP (49, 123). When evaluating pulmonary involvement radiologically, it is important to apply the protocol for ILDs, which involves the use of a HRCT scan (with thin sections, ≤ 1.5 mm rather than 10 mm). Scans in prone decubitus are required to differentiate the areas of gravitational hyperdensity from pathological changes in the density of the lung parenchyma. In addition, expiratory scans are required to rule out the presence of air entrapment (124).

**Figure 6:**
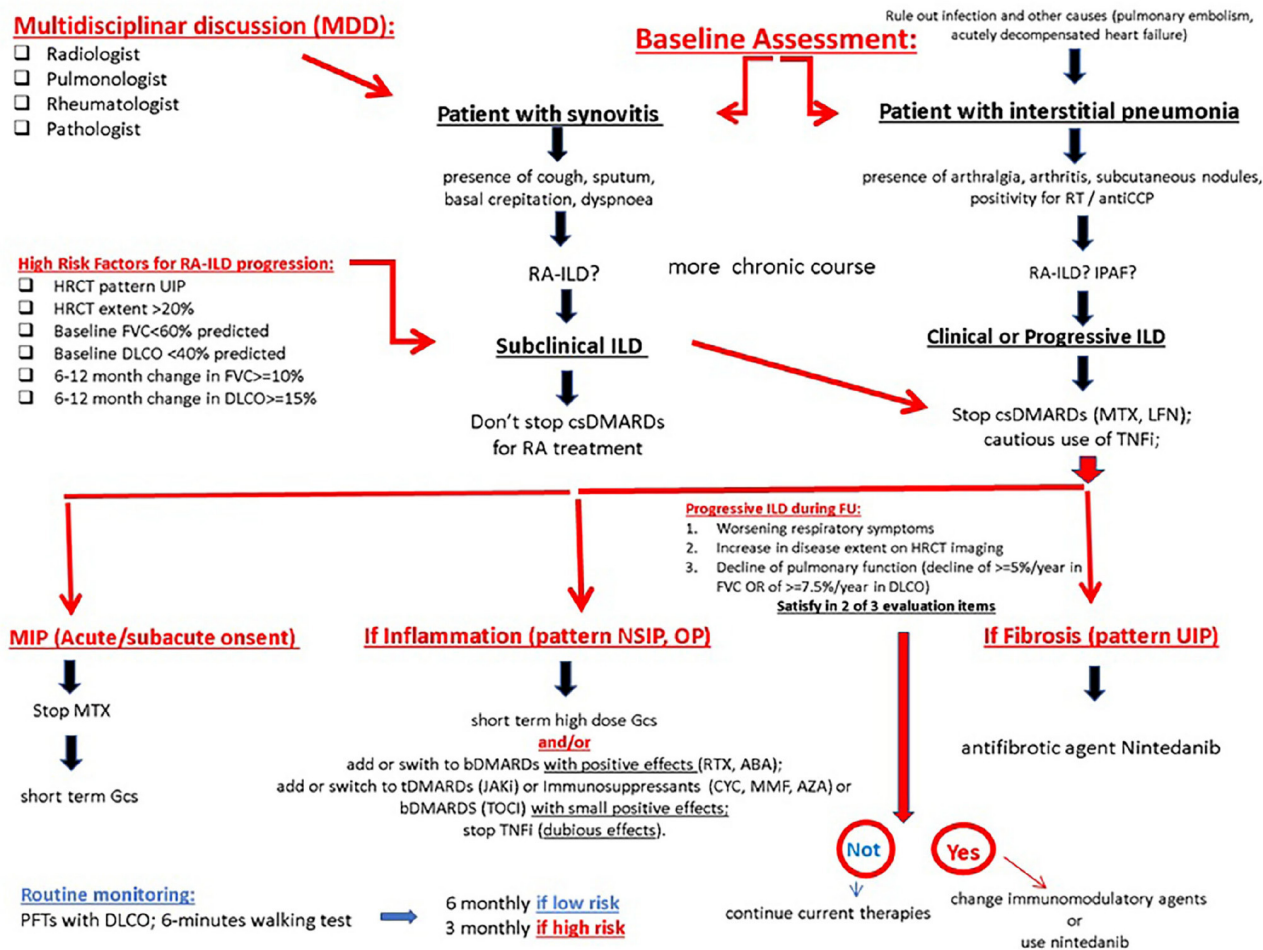
The proposed algorithm for pulmonary symptoms in RA. ABA, abatacept; bDMARD, biological disease-modifying antirheumatic drug; csDMARD, conventional synthetic disease-modifying antirheumatic drug; ILD, interstitial lung disease; MTX, methotrexate; MTX-pneu, MTX-pneumonitis; RA, rheumatoid arthritis; RTX, rituximab; TCZ, tocilizumab. MIP Methotrexate-induced pneumonia; GCs glucocorticoids.

In a recent study, Yamakawa et al. take the stock of the current evidence regarding firstly the assessment and secondarily the treatment of ILD-RA. So, the author propose a focus on the risk assessment before the initiation of a biologic therapy, and disease monitoring during treatment (125) (Figure 6). The gold standard for diagnosing RA-ILD involves a comprehensive and multidisciplinary discussion of clinical history, clinical examination, blood testing, PFTs, HRCT, and lung biopsy when available. Although a multidisciplinary discussion of newly detected ILDs most often includes pulmonologists, radiologists, and pathologists, the inclusion of rheumatologists would critically improve the identification of CTD-ILDs (126). As the first RA manifestation may be inflammatory arthritis as well as ILD in patients who subsequently develop RA-ILD (57, 127), both rheumatologists and pulmonologists have an important role in disease detection and evaluation (Figure 6). The high prevalence of subclinical ILD on HRCT scans of patients with RA demonstrates that a screening approach relying on clinical signs and symptoms may not be sufficiently sensitive in detecting RA-ILD (128, 129). To date, no biomarkers have been validated in clinical practice to help clinicians diagnose subclinical RA-ILD. However, in the presence of short breath, dry cough, crackles, clubbing, and extra-articular RA manifestations (i.e., subcutaneous nodules), it is useful to perform PFTs and HRCT to examine a possible RA-ILD. When ILD is the initial manifestation, clinicians should test the hypothesis of RA as a potential underlying cause, differentiating it from other CTD-ILDs and IIPs. In this regard, the investigation of an autoimmune history or familiarity, clinical examination of the joints, dosage of RA-specific antibodies (RF and ACPAs), and the search of an UIP pattern at HRCT are strongly advised. Although ACPAs are highly specific for RA (130), they may also be positive in the setting of chronic lung diseases even in the absence of RA, as occurring in IPAF (54). On the other hand, individuals with ACPAs but without inflammatory arthritis are at a high risk of developing RA later (131). When the diagnosis of RA-ILD is made, it is important not to overlook conservative and supportive care in these patients. Conservative intervention involves the prevention of lung infections through antibiotic prophylaxis and vaccinations; the management of comorbidities such as COPD or GERD; the cessation of cigarette smoking; the use of supplemental oxygen in case of desaturation; and pulmonary rehabilitation. Last but not the least, the inclusion of severe and refractory patients on the transplant lists should be considered (132). In the presence of subclinical ILD, a continuation of the DMARD used for controlling arthritis according to the current recommendations for RA management is advised. In the presence of a clinically overt and/or progressive ILD, the discontinuation of DMARDs other than MTX with potential pulmonary toxicity (e.g., leflunomide and sulfasalazine) should be considered, while alternative DMARDs (e.g., cyclophosphamide, MMF, or azathioprine), in combination with glucocorticoids, may be preferred. Juge et al. recently suggest that the use of MTX is not associated with an increased risk of RA-ILD in patients with RA, and that ILD was detected later in MTX-treated patients. So, the therapy with MTX can be conditionally recommended for patients with RA-ILD (75). Otherwise, TNFi should be used with caution or reconsidered in these cases although the evidence for the harm of TNFi is weak (133). In the presence of clinical, functional, and/or radiological worsening despite treatment, further therapeutic changes are suggested, particularly, the introduction of biologics that have shown to improve or stabilize pulmonary disease (e.g., abatacept, rituximab, or tocilizumab) or the introduction of anti-fibrotic agents (e.g., nintedanib) in the presence of high fibrotic scores on the HRCT scan (132). Treatment will be tailored also taking into account the predominance of joint or respiratory symptoms (133). Therapeutic flowcharts for RA-ILD certainly differ from those used for IPF, for which glucocorticoids are not indicated and the first-choice drugs are anti-fibrotic (including pirfenidone) (74) (Figure 6).

In conclusion, a comprehensive and multidisciplinary approach is required for differential diagnosis of ILDs, early identification of RA-ILD, and timely intervention on progressive and fibrosing forms. It is especially crucial to contextualize any respiratory symptoms in a given patient with RA. In the setting of a recently introduced MTX treatment, MTX pulmonary toxicity is certainly a concern, particularly if the onset of respiratory symptoms is acute or subacute; in such cases, MTX needs to be stopped and high-dose glucocorticoids, along with supportive care, should be timely started. If the onset of respiratory symptoms is instead more insidious, RA-ILD is more likely to occur, and immunotherapies with csDMARDS and/or bDMARDs should be readjusted based on the severity of lung and joint disease and possible comorbidities. Ongoing investigations and future RCTs will better clarify the strategies to be put in place for the optimal management of RA-ILD.

## Author Contributions

AL: drafting. AML, MM, DM, and KR: data collection. GZ: review of literature. EZ: review of the manuscript. PF: manuscript drafting. AM: supervisor. All authors contributed to the article and approved the submitted version.

## Conflict of Interest

The authors declare that the research was conducted in the absence of any commercial or financial relationships that could be construed as a potential conflict of interest.

## Publisher's Note

All claims expressed in this article are solely those of the authors and do not necessarily represent those of their affiliated organizations, or those of the publisher, the editors and the reviewers. Any product that may be evaluated in this article, or claim that may be made by its manufacturer, is not guaranteed or endorsed by the publisher.

## Footnotes

^1^Available online at: https://www.fda.gov/news-events/press-announcements/fda-approves-first-treatment-group-progressive-interstitial-lung-diseases.

## References

[1] Cortet B Perez T Roux N Flipo RM Duquesnoy B Delcambre B. Pulmonary function tests and high resolution computed tomography of the lungs in patients with rheumatoid arthritis. Ann Rheum Dis. (1997) 56:596–600. 10.1136/ard.56.10.596 9389220 PMC1752273

[2] Kanat F Levendoglu F Teke T. Radiological and functional assessment of pulmonary involvement in the rheumatoid arthritis patients. Rheumatol Int. (2007) 27:459–66. 10.1007/s00296-006-0234-0 17028857

[3] Brown KK. Rheumatoid lung disease. Proc Am Thorac Soc. (2007) 4:443–8. 10.1513/pats.200703-045MS 17684286 PMC2647595

[4] Yunt ZX Solomon JJ. Lung disease in rheumatoid arthritis. Rheum Dis Clin North Am. (2015) 41:225–36. 10.1016/j.rdc.2014.12.004 25836639 PMC4415514

[5] Olson AL Swigris JJ Sprunger DB Fischer AR Fernandez-Perez E Solomon J. Rheumatoid arthritis-interstitial lung disease-associated mortality. Am J Respir Crit Care Med. (2011) 183:372–8. 10.1164/rccm.201004-0622OC 20851924 PMC5450769

[6] Castelino FV Varga J. Interstitial lung disease in connective tissue diseases: evolving concepts of pathogenesis and management. Arthritis Res Ther. (2010) 12:213. 10.1186/ar3097 20735863 PMC2945045

[7] Bongartz T Nannini C Yimy F Velasquez M Achenbach SJ Crowson CS. Incidence and Mortality of Interstitial Lung Disease in Rheumatoid Arthritis A Population-Based Study. Arthritis Rheumatism. (2010) 62:1583–91. 10.1002/art.27405 20155830 PMC4028137

[8] Gizinski AM Mascolo M Loucks JL Kervitsky A Meehan RT Brown KK. Rheumatoid arthritis (RA)-specific autoantibodies in patients with interstitial lung disease and absence of clinically apparent articular RA. Clin Rheumatol. (2009) 28:611–3. 10.1007/s10067-009-1128-9 19252818 PMC4084723

[9] De Lauretis A Veeraraghavan S Renzoni E. Review series: aspects of interstitial lung disease: connective tissue disease-associated interstitial lung disease: how does it differ from IPF? How should the clinical approach differ? Chronic Respir Dis. (2011) 8:53–82. 10.1177/1479972310393758 21339375

[10] American Thoracic Society European Respiratory Society. American Thoracic Society/European Respiratory Society international multidisciplinary consensus classification of the idiopathic interstitial pneumonias. Am J Respir Crit Care Med. (2002) 165:277–304. 10.1164/ajrccm.165.2.ats01 11790668

[11] Wilczynska MM Condliffe AM McKeon DJ. Coexistence of bronchiectasis and rheumatoid arthritis: revisited. Respir Care. (2013) 58:694–701. 10.4187/respcare.01857 22782500

[12] De Soyza A McDonnell MJ Goeminne PC Aliberti S Lonni S Davison J. Bronchiectasis rheumatoid overlap syndrome (BROS) is an independent risk factor for mortality in patients with bronchiectasis: a multicentre cohort study. Chest. (2017) 151:1247–54. 10.1016/j.chest.2016.12.024 28093268

[13] Mori S Koga Y Sugimoto M. Different risk factors between interstitial lung disease and airway disease in rheumatoid arthritis. Respir Med. (2012) 106:1591–9. 10.1016/j.rmed.2012.07.006 22867979

[14] Assayag D Lubin M Lee JS King TE Collard HR Ryerson CJ. Predictors of mortality in rheumatoid arthritis-related interstitial lung disease. Respirology. (2014) 19:493–500. 10.1111/resp.12234 24372981

[15] Nannini C Medina-Velasquez YF Achenbach SJ Crowson CS Ryu JH Vassallo R. Incidence and mortality of obstructive lung disease in rheumatoid arthritis: a population-based study. Arthritis Care Res. (2013) 65:1243–50. 10.1002/acr.21986 23436637 PMC4017238

[16] Giles JT Danoff SK Sokolove J Wagner C Winchester R Pappaset DA. Association of fine specificity and repertoire expansion of anticitrullinated peptide antibodies with rheumatoid arthritis associated interstitial lung disease. Ann Rheum Dis. (2014) 73:1487–94. 10.1136/annrheumdis-2012-203160 23716070 PMC3883892

[17] Zhu J Zhou Y Chen X Li J. A metaanalysis of the increased risk of rheumatoid arthritis-related pulmonary disease as a result of serum anticitrullinated protein antibody positivity. J Rheumatol. (2014) 41:1282–9. 10.3899/jrheum.131341 24882837

[18] Schernthaner G Scherak O Kolarz G Kummer F. Seropositive rheumatoid arthritis associated with decreased diffusion capacity of the lung. Ann Rheum Dis. (1976) 35:258–62. 10.1136/ard.35.3.258 984906 PMC1006550

[19] Smolen JS Landewe R Bijlsma J Burmester GR Dougados M Kerschbaumer A. EULAR recommendations for the management of rheumatoid arthritis with synthetic and biological disease- modifying antirheumatic drugs: 2019 update. Ann Rheum Dis. (2020) 79:685–99. 10.1136/annrheumdis-2019-216655 31969328

[20] Thun MJ Carter BD Feskanich D Freedman ND Prentice R Lopezet AD. 50-year trends in smoking-related mortality in the United States. N Engl J Med. (2013) 368:351–64. 10.1056/NEJMsa1211127 23343064 PMC3632080

[21] Chatzidionisyou A Catrina IA. The lung in rheumatoid arthritis, cause or consequence? Review Curr Opin Rheumatol. (2016) 28:76–82. 10.1097/BOR.0000000000000238 26599384

[22] Feraco P Bazzocchi A Righi S Zampogna G Savastio G Salizzoni S. “Involvement of cricoarytenoid joints in rheumatoid arthritis,” JCR: Journal of Clinical Rheumatology. (2009) 15:264. 10.1097/RHU.0b013e3181b2a965 19654493

[23] Abe K Mitsuka T Yamaoka Keishi Yamashita A Masaomi Yamashita A Norimoto M. Sudden glottic stenosis caused by cricoarytenoid joint involvement due to rheumatoid arthritis. Intern Med. (2013) 52:2469–72. 10.2169/internalmedicine.52.0882 24190155

[24] Berjawi GI Uthman I Mahfoud L Tanbouzi Husseini S Nassar J Kotobi A. “Cricothyroid joint abnormalities in patients with rheumatoid arthritis. J Voice. (2010) 24:732–7. 10.1016/j.jvoice.2009.06.005 20335001

[25] Shaw M Collins F Ho A Raghu G. Rheumatoid arthritis-associated lung disease. Eur Respir Rev. (2015) 24:1–16. 10.1183/09059180.00008014 25726549 PMC9487778

[26] Devouassoux G Cottin V Liote H Frachon I Schuller A Béjui-Thivoletet F. Characterisation of severe obliterative bronchiolitis in rheumatoid arthritis. Eur Respir J. (2009) 33:1053–61. 10.1183/09031936.00091608 19129282

[27] Penny WJ Knight RK Rees AM Thomas AL Smith AP. Obliterative bronchiolitis in rheumatoid arthritis. Ann Rheum Dis. (1982) 41:469–72. 10.1136/ard.41.5.469 7125715 PMC1001024

[28] Izumiyama T Hama H Miura M Suzuki Y Sawai T Saito T. Frequency of broncho-bronchiolar disease in rheumatoid arthritis: An examination by high-resolution computed tomography. Mod Rheumatol. (2002) 12:311–7. 10.1007/s101650200055 24383998

[29] Balbir-Gurman A Yigla M Nahir AM. Braun- Moscovici Y. Rheumatoid Pleural Effusion. Semin Arthritis Rheum. (2006) 35:368–78. 10.1016/j.semarthrit.2006.03.002 16765714

[30] Bouros D Pneumatikos i Tzouvelekis A. Pleural involvement in systemic autoimmune disorders. Respiration. (2008) 75:361–71. 10.1159/000119051 18477860

[31] English JC Leslie KO. Pathology of the Pleura. Clin Chest Med. (2006) 27:157–80. 10.1016/j.ccm.2006.01.006 16716811

[32] Henriet AC Diot E Marchand-Adam S. Organising pneumonia can be the inaugural manifestation in connective tissue diseases, including sjogren's syndrome. Eur Respir Rev. (2010) 19:161–3. 10.1183/09059180.00002410 20956186 PMC9682575

[33] Dalvi V Gonzalez EB Lovett L. Lymphocytic interstitial pneumonitis (LIP) in Sjogren's syndrome: a case report and a review of the literature. Clin Rheumatol. (2007) 26:1339–43. 10.1007/s10067-006-0351-x 16897120

[34] Ziff M. The rheumatoid nodule. Arthritis Rheum. (1990) 33:761–7. 10.1002/art.1780330601 2194460

[35] Galiè N Humbert M Vachiery JL Gibbs S Lang I Torbicki A. 2015 ESC/ERS Guidelines for the diagnosis and treatment of pulmonary hypertension:The Joint Task Force for the Diagnosis and Treatment of PulmonaryHypertension of the European Society of Cardiology (ESC) and the European Respiratory Society (ERS): Endorsed by: Association for European Paediatric and Congenital Cardiology (AEPC), International Society forHeart and Lung Transplantation (ISHLT). Eur Heart J. (2016) 37:67–119. 10.1093/eurheartj/ehv317 26320113

[36] Urisman A Jones KD. Pulmonary pathology in connective tissue disease. Seminars Respir Crit Care Med. (2014) 35:201–12. 10.1055/s-0034-1371543 24668535

[37] Wells AU Denton CP. Interstitial lung disease in connective tissue disease—mechanisms and management. Nat Rev Rheumatol. (2014) 10:728–39. 10.1038/nrrheum.2014.149 25266451

[38] Wallace B Vummidi D Khanna D. Management of connective tissue diseases associated interstitial lung disease: A review of the published literature. Curr Opin Rheumatol. (2016) 28:236–45. 10.1097/BOR.0000000000000270 27027811 PMC4826478

[39] Park JH Kim DS Park IN Jang SJ Kitaichi M Nicholson AG. Prognosis of fibrotic interstitial pneumonia: idiopathic versus collagen vascular disease-related subtypes. Am J Respir Crit Care Med. (2007) 175:705–11. 10.1164/rccm.200607-912OC 17218621

[40] Sakai F Noma S Kurihara Y. Leflunomide-related lung injury in patients with rheumatoid arthritis: Imaging features. Mod Rheumatol. (2005) 15:173–9. 10.3109/s10165-005-0387-9 17029058

[41] Bartels CM Bell CL Shinki K Rosenthal A Bridges AJ. Changing trends in serious extra-articular manifestations of rheumatoid arthritis among united state veterans over 20 years. Rheumatology. (2010) 49:1670–5. 10.1093/rheumatology/keq135 20463190 PMC2919197

[42] Alunno A Gerli R Giacomelli R Carubbi F. Review Article: Clinical, Epidemiological, and Histopathological Features of Respiratory Involvement in Rheumatoid Arthritis. BioMed Res Int. (2017) 2017:7915340. 10.1155/2017/7915340 29238722 PMC5697381

[43] Marcucci E Bartoloni E Alunno A Leone MC Cafaro G Luccioli F. VExtra-articular rheumatoid arthritis. REVIEW Reumatismo. (2018) 70:212–24. 10.4081/reumatismo.2018.1106 30570239

[44] Saag KG Teng GG Patkar NM. American College of Rheumatology 2008 recommendations for the use of nonbiologic and biologic diseasemodifying antirheumatic drugs in rheumatoid arthritis. Arthritis Rheum. (2008) 59:762–84. 10.1002/art.23721 18512708

[45] Padley SP Hansell DM Flower CD Jennings P. Comparative accuracy of high resolution computed tomography and chest radiography in the diagnosis of chronic diffuse infiltrative lung disease. Clin Radiol. (1991) 44:222–6 10.1016/S0009-9260(05)80183-7 1959296

[46] Tanaka N Kim JS Newell JD Brown KK Cool CD Meehan R. Rheumatoid arthritis-related lung diseases: CT findings. Radiology. (2004) 232:81–91. 10.1148/radiol.2321030174 15166329

[47] Solomon JJ Ryu JH Tazelaar HD Myers JL Tuder R Cool CD. Fibrosing interstitial pneumonia predicts survival in patients with rheumatoid arthritis-associated interstitial lung disease (RA-ILD). Respir Med. (2013) 107:1247–52. 10.1016/j.rmed.2013.05.002 23791462

[48] Swensen SJ Aughenbaugh GL Myers JL. Diffuse lung disease: diagnostic accuracy of CT in patients undergoing surgical biopsy of the lung. Radiology. (1997) 205:229–34. 10.1148/radiology.205.1.9314990 9314990

[49] Kim EJ Elicker BM Maldonado F Webb WR RyuJ H Van Udenet JH. Usual interstitial pneumonia in rheumatoid arthritis-associated interstitial lung disease. Eur Respir J. (2010) 35:1322–8. 10.1183/09031936.00092309 19996193

[50] Nurmi HM Purokivi MK Karkkainen MS Kettunen AP Selander TA Kaarteenaho RL. Variable course of disease of rheumatoid arthritis associated usual interstitial pneumonia compared to other subtypes. BMC Pulm Med. (2016) 16:107. 10.1186/s12890-016-0269-2 27461264 PMC4962382

[51] Mori S Cho I Koga Y. A simultaneous onset of organizing pneumonia and rheumatoid arthritis, along with a review of the literature. Mod Rheumatol. (2008) 18:60–6. 10.3109/s10165-007-0004-1 18159567

[52] Lee HK Kim DS Yoo B Joon Beom S Jae-Yoon R Colby TV. Histopathologic pattern and clinical features of rheumatoid arthritis-associated interstitial lung disease. Chest. (2005) 127:2019–27. 10.1378/chest.127.6.2019 15947315

[53] Assayag D Lee JS King TE Jr. Rheumatoid arthritis associated interstitial lung disease: a review. Medicina (B Aires). (2014) 74:158–65. 24736263

[54] Fischer A Solomon JJ du Bois RM. Lung disease with anti-CCP antibodies but not rheumatoid arthritis or connective tissue disease. Respir Med. (2012) 106:1040–7. 10.1016/j.rmed.2012.03.006 22503074 PMC3753791

[55] Brannan HM Good CA Divertie MB. Pulmonary disease associated with rheumatoid arthritis. JAMA. (1964) 189:914–8. 10.1001/jama.1964.03070120036009 14174328

[56] Mascolo M Loucks J Gizinski A. Rheumatoid arthritis (RA)-specific autoantibodies in patients with interstitial lung disease and absence of clinically apparent articular RA. Clin Rheumatol. (2008) 15:231–3. 19252818 10.1007/s10067-009-1128-9 PMC4084723

[57] Gabbay E Tarala R Will R Carrol G Adler B Cameron D. Interstitial lung disease in recent onset rheumatoid arthritis. Am J Respir Crit Care Med. (1997) 156:528–35. 10.1164/ajrccm.156.2.9609016 9279235

[58] Chen J Shi Y Wang X Huang H Ascherman D. Asymptomatic preclinical rheumatoid arthritis-associated interstitial lung disease. Clin Dev Immunol. (2013) 2013:406927. 10.1155/2013/406927 23983768 PMC3747456

[59] Hamblin MJ Horton MR. Rheumatoid arthritisassociated interstitial lung disease: diagnostic dilemma. Pulm Med. (2011) 2011:872120. 10.1155/2011/872120 21660199 PMC3109679

[60] Saag KG Kolluri S Koehnke RK Georgou TA Rachow JW Hunninghake GW. Rheumatoid arthritis lung disease. Determinants of radiographic and physiologic abnormalities. Arthritis Rheum. (1996) 39:1711–9. 10.1002/art.1780391014 8843862

[61] Mohd Noor N Mohd Shahrir MS Shahid MS Abdul Manap R Shahizon Azura AM Azhar Shah S. Clinical and high resolution computed tomography characteristics of patients with rheumatoid arthritis lung disease. Int J Rheum Dis. (2009) 12:136–44. 10.1111/j.1756-185X.2009.01376.x 20374331

[62] Pappas DA Giles JT Connors G Lechtzin N Bathon JM Danoff SK. Respiratory symptoms and disease characteristics as predictors of pulmonary function abnormalities in patients with rheumatoid arthritis: an observational cohort study. Arthritis Res Ther. (2010) 12:R104. 10.1186/ar3037 20507627 PMC2911894

[63] Yousem SA Colby TV Carrington CB. Lung biopsy in rheumatoid arthritis. Am Rev Respir Dis. (1985) 131:770–7. 3873887 10.1164/arrd.1985.131.5.770

[64] Koslow M Young JR Yi ES Baqir M Decker PA Johnson GB. Rheumatoid pulmonary nodules: clinical and imaging features compared with malignancy. Eur Radiol. (2019) 29:1684–92. 10.1007/s00330-018-5755-x 30288558

[65] Akiyama N Toyoshima M Kono M Nakamura Y Funai K Suda T. Methotrexate-induced accelerated pulmonary nodulosis. Am J Respir Crit Care Med. (2015) 192:252–3 10.1164/rccm.201502-0364IM 26177173

[66] Hill AT Sullivan AL Chalmers JD De Soyza A Elborn SJ Floto AR. British Thoracic Society Guideline forbronchiectasis in adults. Thorax. (2019) 74:1–69. 10.1136/thoraxjnl-2018-212463 30545985

[67] Habib HM Eisa AA Arafat WR Mohammed AM. Pulmonary involvement in early rheuma-toid arthritis patients. Clin Rheumatol. (2011) 30:217–21. 10.1007/s10067-010-1492-5 20503061

[68] Duarte AC Porter J Leandro MJ. Bronchiectasis in rheumatoid arthritis. a clinical appraisial. Joint Bone Spine. (2020) 87:419–24. 10.1016/j.jbspin.2019.12.006 32007647

[69] Singh JA Saag KG Bridges SL Jr Bannuru R Akl EA Osani M. 2015 American College of Rheumatology guideline for the treatment of rheumatoid arthritis. Arthritis Care Res. (2016) 68:1–25. 10.1002/acr.22783 26545825

[70] Song JW Lee HK Lee CK Chae EJ Jang SJ Colby TV. Clinical course and outcome of rheumatoid arthritis-related usual interstitial pneumonia. Sarcoidosis Vasc Diffuse Lung Dis. (2013) 30:103–12. 24071881

[71] Zamora-Legoff JA Krause ML Crowson CS Ryu JH Matteson EL. Risk of serious infection in patients with rheumatoid arthritis-associated interstitial lung disease. Clin Rheumatol. (2016) 35:2585–9. 10.1007/s10067-016-3357-z 27448151

[72] Wells AU Hirani N on behalf of the British Thoracic Society Interstitial Lung Disease Guideline Group a a subgroup of the British Thoracic Society Standards of Care Committee in in collaboration with the Thoracic Society of Australia and New Zealand and the Irish Thoracic Society. Interstitial lung disease guideline: the British Thoracic Society in collaboration with the Thoracic Society of Australia and New Zealand and the Irish Thoracic Society. Thorax. (2008) 63(Suppl V):v1–v58. 10.1136/thx.2008.101691 18757459

[73] Kim EJ Collard HR King TE Jr. Rheumatoid arthritis-associated interstitial lung disease: the relevance of histopathologic and radiographic pattern. Chest. (2009) 136:1397–405. 10.1378/chest.09-0444 19892679 PMC2818853

[74] Fragoulis GE Nikiphorou E Larsen J Korsten P Conway R. Methotrexate-Associated Pneumonitis and Rheumatoid Arthritis-Interstitial Lung Disease: Current Concepts for the Diagnosis and Treatment. Front Med. (2019) 6:238. 10.3389/fmed.2019.00238 31709258 PMC6819370

[75] Juge PA Lee JS Lau J Kawano-Dourado L Rojas Serrano J Sebastiani M. Methotrexate and rheumatoid arthritis associated interstitial lung disease. Eur Respir J. (2021) 57:2000337. 10.1183/13993003.00337-2020 32646919 PMC8212188

[76] Kiely P Busby AD Nikiphorou E Sullivan K Walsh DA Creamer P. Is incident rheumatoid arthritis interstitial lung disease associated with methotrexate treatment? Results from a multivariate analysis in the ERAS and ERAN inception cohorts. BMJ Open. (2019) 9:e028466. 10.1136/bmjopen-2018-028466 31061059 PMC6501950

[77] Kur-Zalewska J Kisiel B Kania-Pudło M Tłustochowicz M Chciałowski A Tłustochowicz W. A dose-dependent beneficial effect of methotrexate on the risk of interstitial lung disease in rheumatoid arthritis patients. PLoS ONE. (2021) 16:e0250339. 10.1371/journal.pone.0250339 33861812 PMC8051807

[78] Fischer A Brown KK Du Bois RM Frankel SK Cosgrove GP Fernandez-Perez ER. Mycophenolate mofetil improves lung function in connective tissue disease-associated interstitial lung disease. J Rheumatol. (2013) 40:640–6. 10.3899/jrheum.121043 23457378 PMC3676865

[79] Tashkin DP Elashoff R Clements PJ Goldin J Roth MD Furst DE. Cyclophosphamide versus placebo in scleroderma lung disease. N Engl J Med. (2006) 354:2655–66. 10.1056/NEJMoa055120 16790698

[80] Tashkin DP Roth MD Clements PJ Furst DE Khanna D Kleerup EC. Mycophenolate mofetil versus oral cyclophosphamide in scleroderma-related interstitial lung disease (SLS II): a randomised controlled, double-blind, parallel group trial. Lancet Respir Med. (2016) 4:708–19. 10.1016/S2213-2600(16)30152-7 27469583 PMC5014629

[81] Oldham JM Lee C Valenzi E Witt LJ Adegunsoye A Hsu S. Azathioprine response in patients with fibrotic connective tissue disease-associated interstitial lung disease. Respir Med. (2016) 121:117–22. 10.1016/j.rmed.2016.11.007 27888985 PMC5134419

[82] Chang HK Park W Ryu DS. Successful treatment of progressive rheumatoid interstitial lung disease with cyclosporine: a case report. J Korean Med Sci. (2002) 17:270–3. 10.3346/jkms.2002.17.2.270 11961317 PMC3054856

[83] Yamano Y Taniguchi H Kondoh Y Ando M Kataoka K Taiki H. Multidimensional improvement in connective tissue disease-associated interstitial lung disease: two courses of pulse dose methylprednisolone followed by low-dose prednisone and tacrolimus. Respirology. (2018) 23:1041–8. 10.1111/resp.13365 30011421

[84] Roubille C Haraoui B. Interstitial lung diseases induced or exacerbated by DMARDS and biologic agents in rheumatoid arthritis: a systematic literature review. Semin Arthritis Rheum. (2014) 43:613–26. 10.1016/j.semarthrit.2013.09.005 24231065

[85] Parry SD Barbatzas C Peel ET Barton JR. Sulphasalazine and lung toxicity. Eur Respir J. (2002) 19:756–64. 10.1183/09031936.02.00267402 11999006

[86] Ulubaş B Sahin G Ozer C Aydin O Ozgür E Apaydin D. Bronchiolitis obliterans organizing pneumonia associated with sulfasalazine in a patient with rheumatoid arthritis. Clin Rheumatol. (2004) 23:249–51 10.1007/s10067-003-0848-5 15168156

[87] Sawada T Inokuma S Sato T Otsuka T Saeki Y Takeuchi T. Study Committee for Leflunomide-induced Lung Injury, Japan College of Rheumatology. Leflunomide-induced interstitial lung disease: prevalence and risk factors in Japanese patients with rheumatoid arthritis. Rheumatology (Oxford). (2009) 48:1069–72. 10.1093/rheumatology/kep052 19321513

[88] Hyeon J Sung-Il K Jun-Hee L Sang-Il L Wan-Hee Y Jung-Yoon C. Risk of interstitial lung disease associated with leflunomide treatment in Korean patients with rheumatoid arthritis. Arthritis Rheum. (2007) 56:2094–6. 10.1002/art.22666 17530652

[89] Jeurissen ME Boerbooms AM van de Putte LB Jeurissen ME de Graaf R Mulder J. Methotrexate versus azathioprine in the treatment of rheumatoid arthritis A forty-eight-week randomized, double-blind trial. Arthritis Rheum. (1991) 34:961–72. 10.1002/art.1780340805 1859490

[90] Willkens RF Urowitz MB Stablein DM Fudman EK Fiechtner JJ Hudson NP. Comparison of azathioprine, methotrexate, and the combination of both in the treatment of rheumatoid arthritis. A controlled clinical trial. Arthritis Rheum. (1992) 35:849–56. 10.1002/art.1780350802 1642652

[91] Perez-Alvarez R. Perez-de-Lis M Diaz-Lagares C Pego-Reigosa JM Retamozo S Bove A. Interstitial lung disease induced or exacerbated by TNF-targeted therapies: analysis of 122 cases. Semin Arthritis Rheum. (2011) 41:256–64. 10.1016/j.semarthrit.2010.11.002 21277618

[92] Nakashita T Ando K Kaneko N Takahashi K Motojima S. Potential risk of TNF inhibitors on the progression of interstitial lung disease in patients with rheumatoid arthritis. BMJ Open. (2014) 4:e005615. 10.1136/bmjopen-2014-005615 25125479 PMC4139628

[93] Dixon WG Hyrich KL Watson KD Lunt M. Influence of anti-TNF therapy on mortality in patients with rheumatoid arthritis-associated interstitial lung disease: results from the British Society for Rheumatology Biologics Register. Ann Rheum Dis. (2010) 69:1086–91. 10.1136/ard.2009.120626 20444754 PMC2935328

[94] Curtis JR Sarsour K Napalkov P Costa LA Schulman KL. Incidence and complications of interstitial lung disease in users of tocilizumab, rituximab, abatacept and antitumor necrosis factor alpha agents, a retrospective cohort study. Arthritis Res Ther. (2015) 17:319. 10.1186/s13075-015-0835-7 26555431 PMC4641344

[95] Kang EH Jin Y Desai RJ. Risk of exacerbation of pulmonary comorbidities in patients with rheumatoid arthritis after initiation of abatacept versus TNF inhibitors: a cohort study. Semin Arthritis Rheum. (2020) 50:401–8. 10.1016/j.semarthrit.2019.11.010 31813561

[96] Jani M Hirani N Matteson EL Dixon WG. The safety of biologic therapies in RA-associated interstitial lung disease. Nat Rev Rheumatol. (2014) 10:284–94. 10.1038/nrrheum.2013.197 24366321

[97] Druce KL Iqbal K Watson KD Symmons DPM Hyrich KL Kelly C. Mortality in patients with interstitial lung disease treated with rituximab or TNFi as a first biologic. RMD Open. (2017) 3:e000473. 10.1136/rmdopen-2017-000473 28955489 PMC5604605

[98] Huang Y Lin W Chen Z Wang Y Huang Y Tu S. Effect of tumor necrosis factor inhibitors on interstitial lung disease in rheumatoid arthritis: angel or demon? Drug Des Dev Ther. (2019) 13:2111–25. 10.2147/DDDT.S204730 31308625 PMC6616146

[99] Matteson EL Bongartz T Ryu JH. Open-label, pilot study of the safety and clinical effects of rituximab in patients with rheumatoid arthritis-associated interstitial pneumonia. Open J Rheumatol Autoimmune Dis. (2012) 2:53. 10.4236/ojra.2012.23011

[100] Fernandez-Diaz C Loricera J Castaneda S Raquel López-Mejías Clara Ojeda-García Alejandro Olivé. Abatacept in patients with rheumatoid arthritis and interstitial lung disease: a national multicenter study of 63 patients. Semin Arthritis Rheum. (2018) 48:22–7. 10.1016/j.semarthrit.2017.12.012 29422324

[101] Hidalgo-Calleja C Lopez-Sanchez R Fernandez-Aguado SC Fernandez-Lopez J Castro-Oreiro S Serrano-Garcia S. Abatacept in interstitial lung disease associated with rheumatoid arthritis: national multicenter study of 263 patients. Rheumatology. (2020) 59:3906–16. 10.1093/rheumatology/keaa621 33068439

[102] Cassone G Manfredi A Atzeni F Venerito V Vacchi C Picerno V. Safety of Abatacept in Italian Patients with Rheumatoid Arthritis and Interstitial Lung Disease: a multicenter retrospective study. J Clin Med. (2020) 9:277. 10.3390/jcm9010277 31963908 PMC7019755

[103] Vicente-Rabaneda EF Atienza-Mateo B Blanco R Cavagna L Ancochea J Castaneda S. Efficacy and safety of abatacept in interstitial lung disease of rheumatoid arthritis: a systematic literature review. Autoimmun Rev. (2021) 20:102830. 10.1016/j.autrev.2021.102830 33887489

[104] Catarina Duarte A Cordeiro A Miguel Fernandes B Miguel Bernardes M Martins P Cordeiro I. Rituximab in connective tissue disease–associated interstitial lung disease. Clin Rheumatol. (2019) 38:2001–9. 10.1007/s10067-019-04557-7 31016581

[105] Manfredi A Cassone H FFurini F Gremese E VVenerito V Atzeni F. Tocilizumab therapy in rheumatoid arthritis with interstitial lung disease: a multicentre retrospective study. Intern Med J. (2020) 50:1085–90. 10.1111/imj.14670 31661185

[106] Saldarriaga-Rivera LM Lopez-Villegas VJ. Janus kinase inhibitors as a therapeutic option in rheumatoid arthritis and associated interstitial lung disease. Report of four cases. Rev Colomb Reumatol. (2019) 26:137–9. 10.1016/j.rcreue.2018.02.003

[107] Kalyoncu U Bilgin E Erden A Satış H Tufan A Tekgoz E. Tofacitinib in rheumatoid arthritisassociated interstitial lung disease: efficacy and safety analysis from treasure real-life data scientific abstracts EULAR 2021. OP0125. Ann Rheum Dis.

[108] Wollin L Wex E Pautsch A Schnapp G Hostettler KE Stowasser S. Mode of action of nintedanib in the treatment of idiopathic pulmonary fibrosis. Eur Respir J. (2015) 45:1434–45. 10.1183/09031936.00174914 25745043 PMC4416110

[109] Distler O Highland KB Gahlemann M Azuma A Fischer A Mayes MD. Nintedanib for systemic sclerosis-associated interstitial lung disease. N Engl J Med. (2019) 380:2518–28. 10.1056/NEJMoa1903076 31112379

[110] Crestani B Huggins JT Kaye M Costabel U Glaspole I Ogura T. Long-term safety and tolerability of nintedanib in patients with idiopathic pulmonary fibrosis: results from the open-label extension study, INPULSIS-ON. Lancet Respir Med. (2019) 7:60–8. 10.1016/S2213-2600(18)30339-4 30224318

[111] Flaherty KR Wells AU Cottin V Devaraj A LF Walsh SLF Inoue Y. Nintedanib in progressive fibrosing interstitial lung diseases. N Engl J Med. (2019) 381:1718–27. 10.1056/NEJMoa1908681 31566307

[112] Wells AU Flaherty KR Brown KK Devaraj A Walsh SLF Inoue Y. Nintedanib in patients with progressive fibrosing interstitial lung diseases-subgroup analyses by interstitial lung disease diagnosis in the INBUILD trial: a randomised, double-blind, placebo-controlled, parallel-group trial. Lancet Respir Med. (2020) 8:453–60. 10.1016/S2213-2600(20)30036-9 32145830

[113] Juge PA Crestani B Dieude' P. Recent advances in rheumatoid arthritis-associated interstitial lung disease. Curr Opin Pulm Med. (2020) 26:477–86. 10.1097/MCP.0000000000000710 32701675

[114] Neumeier A Keith R. Clinical guideline highlights for the hospitalist: The GOLD and NICE Guidelines for the Management of COPD. J Hosp Med. (2020) 15:240–1. 10.12788/jhm.3368 32118561

[115] Raimundo K Solomon JJ Olson AL Kong AM Cole AL Fischer A. Rheumatoid arthritis-interstitial lung disease in the United States: prevalence, incidence, and healthcare costs and mortality. J Rheumatol. (2019) 46:360–9. 10.3899/jrheum.171315 30442831

[116] Wang CH Li CH Hsieh R Fan CY Hsu TC Chang WC. Proton pump inhibitors therapy and the risk of pneumonia: a systematic review and meta-analysis of randomized controlled trials and observational studies. Expert Opin Drug Saf. (2019) 18:163–72. 10.1080/14740338.2019.1577820 30704306

[117] Raghu G Rochwerg B Zhang Y Cuello Garcia CA Azuma A Behr J. An official ATS/ERS/JRS/ALAT clinical practice guideline: treatment of idiopathic pulmonary fibrosis. an update of the 2011 clinical practice guideline. Am J Respir Crit Care Med. (2015) 192:e3–e19. 10.1164/rccm.201506-1063ST 26177183

[118] Ryerson CJ Urbania TH Richeldi L Mooney JJ Lee JS Jones KD. Prevalence and prognosis of unclassifiable interstitial lung disease. Eur Respir J. (2013) 42:750–7. 10.1183/09031936.00131912 23222877

[119] Ley B Ryerson CJ Vittinghoff E Ryu JH Tomassetti S Lee JS. A multidimensional index and staging system for idiopathic pulmonary fibrosis. Ann Intern Med. (2012) 156:684–91. 10.7326/0003-4819-156-10-201205150-00004 22586007

[120] Collard HR Egan JJ Raghu G. An official ATS/ ERS/JRS/ALAT statement: idiopathic pulmonary fibrosis: evidence-based guidelines for diagnosis and management. Am J Respir Crit Care Med. (2011) 183:788–824. 10.1164/rccm.2009-040GL 21471066 PMC5450933

[121] Egan JJ Martinez FJ Wells AU Williams T. Lung function estimates in idiopathic pulmonary fibrosis: the potential for a simple classification. Thorax. (2005) 60:270–3. 10.1136/thx.2004.035436 15790978 PMC1747374

[122] Latsi PI du Bois RM Nicholson AG Nikolakiopoulou A Hansell DM Nicholson AG. Fibrotic idiopathic interstitial pneumonia: the prognostic value of longitudinal functional trends. Am J Respir Crit Care Med. (2003) 168:531–7. 10.1164/rccm.200210-1245OC 12791580

[123] Flaherty KR Thwaite EL Kazerooni EA Gross BH Toews GB Colby TV. Radiological versus histological diagnosis in UIP and NSIP: survival implications. Thorax. (2003) 58:143–8. 10.1136/thorax.58.2.143 12554898 PMC1746568

[124] Raghu G Remy-Jardin M Myers JL Richeldi L Ryerson CJ. On behalf of the American Thoracic Society, European Respiratory Society, Japanese Respiratory Society, and Latin American Thoracic Society Diagnosis of Idiopathic Pulmonary Fibrosis An Official ATS/ERS/JRS/ALAT Clinical Practice Guideline. Am J Respir Crit Care Med. (2018) 198:e44–e68. 10.1164/rccm.201807-1255ST 30168753

[125] Levi Y Israeli-Shani L Kuchuk M Epstein Shochet G Koslow M Shitrit D. Rheumatological assessment is important for interstitial lung disease diagnosis. J Rheumatol. (2018) 45:1509–14. 10.3899/jrheum.171314 30111640

[126] Koduri G Norton S Young A. Nigel Cox Paul Davies Joe Devlin. Interstitial lung disease has a poor prognosis in rheumatoid arthritis: results from an inception cohort. Rheumatology. (2010) 49:1483–9. 10.1093/rheumatology/keq035 20223814

[127] Kelly CA Saravanan V Nisar M. Rheumatoid arthritis-related interstitial lung disease: associations, prognostic factors and physiological and radiological characteristics – a large multicentre UK study. Rheumatology. (2014) 53:1676–82. 10.1093/rheumatology/keu165 24758887

[128] Salaffi F Carotti M Di Carlo M Di Carlo M Fraticelli P Fischetti C. High-resolution computed tomography of the lung in patients with rheumatoid arthritis: Prevalence of interstitial lung disease involvement and determinants of abnormalities. Medicine (Baltimore). (2019) 98:e17088. 10.1097/MD.0000000000017088 31567944 PMC6756733

[129] Nishimura K Sugiyama D Kogata Y Tsuji G Kogata Y Koshiba M. Meta-analysis: diagnostic accuracy of anticyclic citrullinated peptide antibody and rheumatoid factor for rheumatoid arthritis. Ann Intern Med. (2007) 146:797–808. 10.7326/0003-4819-146-11-200706050-00008 17548411

[130] Quirke AM Perry E Cartwright A Kelly C De Soyza A Eggleton P. Bronchiectasis is a model for chronic bacterial infection inducing autoimmunity in rheumatoid arthritis. Arthritis Rheumatol. (2015) 67:2335–42. 10.1002/art.39226 26017630 PMC4832289

[131] England BR Hershberger D. Management issues in rheumatoid arthritis-associated interstitial lung disease. Curr Opin Rheumatol. (2020) 32:255–63. 10.1097/BOR.0000000000000703 32141954 PMC7331796

[132] Wijsenbeek M Cottin V. Spectrum of Fibrotic Lung Diseases. N Engl J Med. (2020) 383:958–68. 10.1056/NEJMra2005230 32877584

[133] Yamakawa H Ogura T. Kameda Kishaba T Iwasawa T Takemura T. Decision-Making Strategy for the Treatment of Rheumatoid Arthritis-Associated Interstitial Lung Disease (RA-ILD). J Clin Med. (3806) 2021:10. 10.3390/jcm10173806 34501253 PMC8432201

